# Tailoring the grooved texture of electrospun polystyrene nanofibers by controlling the solvent system and relative humidity

**DOI:** 10.1186/1556-276X-9-350

**Published:** 2014-07-14

**Authors:** Wanjun Liu, Chen Huang, Xiangyu Jin

**Affiliations:** 1 Key Laboratory of Textile Science & Technology, Ministry of Education, College of Textiles, Donghua University, No. 2999 North Renmin Road, Songjiang, Shanghai 201620, China

**Keywords:** Electrospinning, Polystyrene nanofibers, Grooved texture, Phase separation

## Abstract

In this study, we have successfully fabricated electrospun polystyrene (PS) nanofibers having a diameter of 326 ± 50 nm with a parallel grooved texture using a mixed solvent of tetrahydrofuran (THF) and *N*,*N*-dimethylformamide (DMF). We discovered that solvent system, solution concentration, and relative humidity were the three key factors to the formation of grooved texture and the diameter of nanofibers. We demonstrated that grooved nanofibers with desired properties (e.g., different numbers of grooves, widths between two adjacent grooves, and depths of grooves) could be electrospun under certain conditions. When THF/DMF ratio was higher than 2:1, the formation mechanism of single grooved texture should be attributed to the formation of voids on the jet surface at the early stage of electrospinning and subsequent elongation and solidification of the voids into a line surface structure. When THF/DMF ratio was 1:1, the formation mechanism of grooved texture should be ascribed to the formation of wrinkled surface on the jet surface at the early stage of electrospinning and subsequent elongation into a grooved texture. Such findings can serve as guidelines for the preparation of grooved nanofibers with desired secondary morphology.

## Background

Electrospinning has been regarded as the most effective and versatile technology to produce nanofibrous nonwovens with controlled fiber morphology, dimensions, and functional components from various polymeric materials. Nanofibrous nonwovens have shown excellent porous properties and vast application potential in areas [1,2] such as biomedical research [3], filtration [4], superhydrophobic surfaces [5,6], energy conversion and storage [7,8], reinforcement, sensors, and many others. Depending on the polymer used, polymer solution properties, nozzle structure, collecting mode, and operating conditions, electrospun nanofibers with various morphologies [9] can be obtained including smooth nanofibers with a circular cross section; beaded, ribbon, helical, porous, necklace-like, side-by-side, core/shell, hollow, and firecracker-shaped nanofibers [10]; rice grain-shaped nanocomposites [11]; etc.

Recent progress in electrospinning has greatly expanded the scope of available morphologies and properties for nanofibers, which further contributes to their applications [12-18]. For example, porous materials have been found in widespread applications such as filtration, catalysis, and biomedical research due to their great increase of surface area and porosity of nanofibers [12]; beaded nanofibers have been used to design superoleophobic surfaces by mimicking the surface of a lotus leaf [13]; and core/shell nanofibers have been applied to the control of drug release by maneuvering drug in the core under specific conditions [14].

Previously, we have reported the fabrication of cellulose acetate butyrate (CAB) and PS fibers with a parallel line surface texture via electrospinning using a mixed solvent system consisting of a highly volatile solvent (e.g., acetone) and a nonvolatile organic solvent [15,16]. These grooved fibers have shown a great potential in the area of tissue engineering and superhydrophobic surfaces. However, how to fabricate grooved fibers with controlled diameters and groove properties (e.g., number of grooves, width between two adjacent grooves, and depth of grooves) is still a challenge, which hampers the further development and applications of grooved nanofibers.

PS excels in the production of electrospun fibers with various morphologies. Considerable efforts [12,16,19-22] have been devoted to the investigation of the secondary structures (e.g., porosity on the surfaces, wrinkled surface, interior porosity) of PS fibers. Although PS fibers with small grooved surfaces have been reported in several studies [20,22], none of them demonstrated how to control this secondary texture. Furthermore, the diameter of grooved PS fibers was normally larger than 1 μm [16]. In this work, grooved nanofibers with an average diameter of 326 ± 50 nm were obtained through optimizing the process parameters. By systematically investigating the influence of variables on the secondary morphology of electrospun PS fibers, we singled out that solvent system, solution concentration, and relative humidity were the three most significant factors in determining the generation of the grooved structure of PS fibers and elucidated the formation mechanism of grooved texture.

## Methods

### Chemicals and materials

PS (Mw = 350,000 g/mol) was purchased from Sigma-Aldrich, Inc, St. Louis, MO, USA. Tetrahydrofuran (THF) and *N*,*N*-dimethylformamide (DMF) were purchased from Shanghai Chemical Reagents Co., Ltd, Shanghai, China. All materials were used without further purification.

### Electrospinning

The PS solution was placed into a syringe with an internal diameter of 0.7 mm, which was mounted on a syringe pump (multisyringe pump TS2-60, BaoDing Longer Precision Pump Co., Ltd., Baoding City, China). A high-voltage supplier (supplied by high-voltage direct-current power supply, BGG6-358, BMEI Co., Ltd., Beijing, China) was connected to the syringe needle. In order to obtain grooved nanofibers and investigate the formation mechanism of grooved texture, 20% (*w*/*v*) PS solutions with different THF/DMF volume ratios (6:0, 5:1, 4:1, 3:1, 2:1, 1:1, 1:2, 1:3, 1:4, 1:5, and 0:6); PS solutions at concentrations of 10%, 15%, 25%, and 30% (*w*/*v*) (THF/DMF ratio, 1:1 *v*/*v*); and 10% (*w*/*v*) PS solutions with different THF/DMF volume ratios (6:0, 5:1, 4:1, 3:1, 2:1, 1:2, 1:3, 1:4, 1:5, and 0:6) were electrospun, while relative humidity (RH), collecting distance, feeding rate, and applied voltage were kept at 60%, 15 cm, 1.5 ml/h, and 12 kV, respectively. To fully investigate the formation mechanism of grooved texture, 20% (*w*/*v*) PS solutions with different THF/DMF volume ratios (6:0, 5:1, 4:1, 3:1, 2:1, 1:1, 1:2, 1:3, 1:4, 1:5, and 0:6) and 10% (*w*/*v*) PS solutions (THF/DMF ratio, 1:1 *v*/*v*) were electrospun under the lowest applied voltage (5 kV). Apart from that, 10% (*w*/*v*) PS solution (THF/DMF ratio, 1:1 *v*/*v*) was used as a model to check the effect of other parameters (e.g., relative humidity, applied voltage, collecting distance, feeding rate).

### Characterization

The surface morphology and cross section of the as-spun PS nanofibers were observed under field emission scanning electron microscopy (FE-SEM) (S-4800, Hitachi Ltd., Tokyo, Japan), and then the SEM images were analyzed using image analysis software (Adobe Acrobat X Pro 10.1.2.45).

## Results and discussion

### Preparation of grooved PS fibers

**Figure 1:**
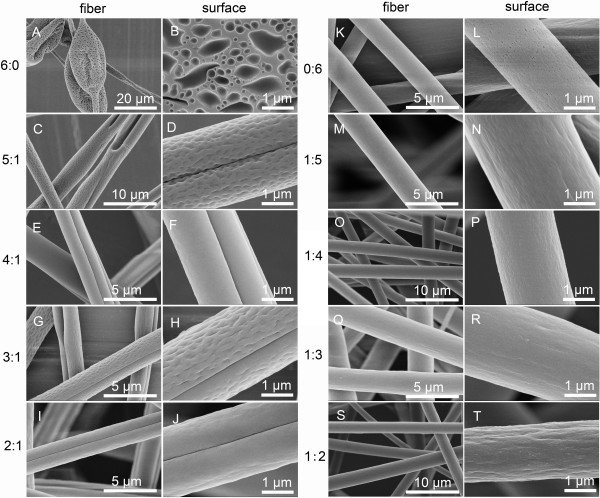
**SEM pictures of fibers and their surfaces fabricated by electrospinning 20% (*w*/*v*) PS solutions with various THF/DMF ratios. (A**, **B)** 6:0, **(C**, **D)** 5:1, **(E**, **F)** 4:1, **(G**, **H)** 3:1, **(I**, **J)** 2:1, **(K**, **L)** 0:6, **(M**, **N)** 1:5, **(O**, **P)** 1:4, **(Q**, **R)** 1:3, and **(S**, **T)** 1:2, *v*/*v*. RH 60%, collecting distance 15 cm, feeding rate 1.5 ml/h, and applied voltage 12 kV.

**Figure 2:**
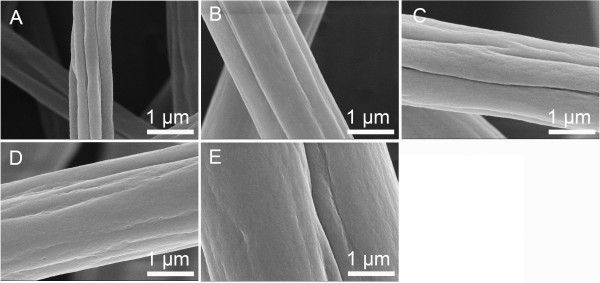
**SEM pictures of grooved PS fibers obtained from different concentrations. (A)** 10% (*w*/*v*), **(B)** 15% (*w*/*v*), **(C)** 20% (*w*/*v*), **(D)** 25% (*w*/*v*), and **(E)** 30% (*w*/*v*). THF/DMF ratio 1:1 *v*/*v*, RH 60%, collecting distance 15 cm, feeding rate 1.5 ml/h, and applied voltage 12 kV.

To explore the effect of solvent system on the secondary morphology of electrospun fibers, 20% (*w*/*v*) PS solutions with various THF/DMF ratios were electrospun (Figures 1 and 2C). Here, it should be noted that PS fibers could be fabricated in a highly stable manner from all PS solutions, except that electrospinning process of 20% (*w*/*v*) PS solution using pure THF as solvent was unstable and often interrupted by the problem of needle clogging. As shown in Figure 1A,B, the resultant beaded fibers from 20% (*w*/*v*) PS/THF solution exhibited a ribbon-like shape which should be attributed to a rapid drying followed by collapse of the liquid jet [21]. In addition, there were numerous big and small pores with irregular shapes on both the surface of beads and fibers. Thermally induced phase separation (TIPS) should be responsible for the porous surface. The evaporation of volatile THF (vapor pressure, 0.36 kPa) absorbed a great amount of heat and cooled the nearby environment; as a result, water vapor began to condense in the vicinity of the jet-air interface. Meanwhile, the positive charges distributed on the surface of the fiber polarized and attracted the condensed tiny water droplets, creating numerous nano- or microscale water pockets, which then dried to generate water imprints or ‘pores’ on the fiber surface [12]. As compared to 20% (*w*/*v*) PS/THF solution, beaded free fibers were obtained from 20% (*w*/*v*) PS/DMF solution, which showed many small elongated pores with an average length of 90 nm (Figure 1K,L).

All the resultant fibers appeared with a deep groove along the axis of PS fibers when the THF/DMF ratio was equal or higher than 2:1 (*v*/*v*) at the concentration of 20% (*w*/*v*) (Figure 1C,D,E,F,G,H,I,J). It should be pointed out that most of these grooved fibers had a wrinkled surface as well as a smooth surface for some. The wrinkled surface is probably due to buckling of a cylindrical polymer shell under compressive radial stresses, arising from the removal of solvent from the core of the jet, and/or a lateral contraction effect from the axial tensile stresses, arising from the continuous stretching of the jet [21]. Interestingly, PS fibers with three to four parallel grooves were fabricated when the THF/DMF ratio was 1:1 (*v*/*v*) (Figure 2C). When the THF/DMF ratio was 1:2 (*v*/*v*), the morphology of obtained fibers showed many small grooves along the axis of PS fibers (Figure 1S,T), while it tend to be smooth when THF/DMF ratio was lower than 1:2 (Figure 1M,N,O,P,Q,R).

**Figure 3:**
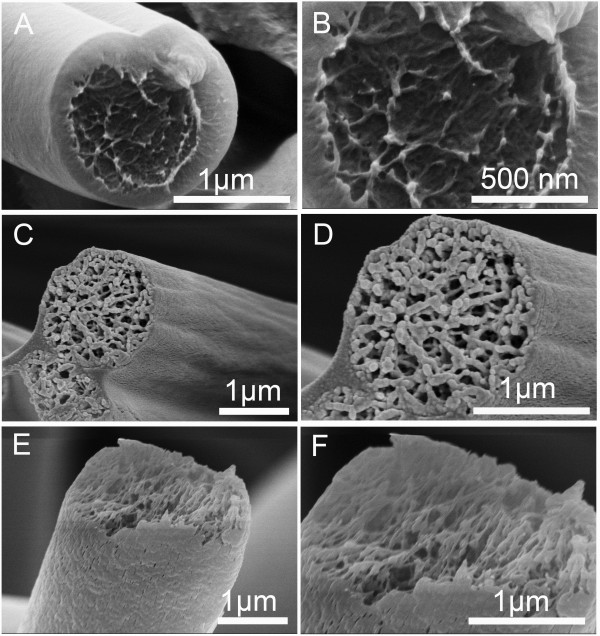
**Cross section of representative fibers.** The fibers were fabricated by electrospinning 20% (*w*/*v*) PS solutions with various THF/DMF ratios. **(A**, **B)** 4:1, **(C**, **D)** 1:1, and **(E**, **F)** 0:6 *v*/*v*. RH 60%, collecting distance 15 cm, feeding rate 1.5 ml/h, and applied voltage 12 kV.

**Figure 4:**
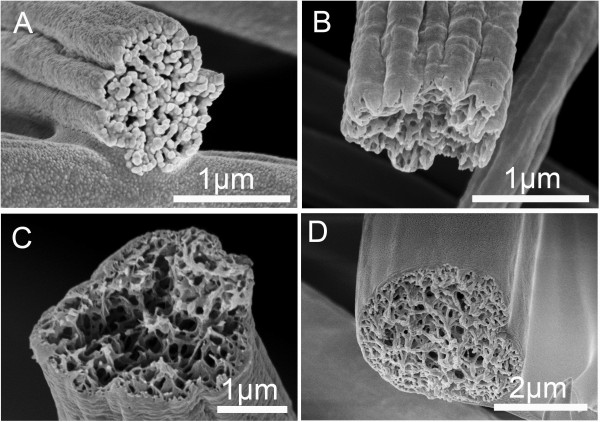
**Cross section of representative PS grooved fibers obtained from different concentrations. (A)** 10% (*w*/*v*), **(B)** 15% (*w*/*v*), **(C)** 25% (*w*/*v*), and **(D)** 30% (*w*/*v*). THF/DMF ratio 1:1 *v*/*v*, RH 60%, collecting distance 15 cm, feeding rate 1.5 ml/h, applied voltage 12 kV.

To investigate the effect of solution concentration, we kept the THF/DMF ratio at 1:1 (*v*/*v*), while the concentration varied from 10% (*w*/*v*) to 30% (*w*/*v*). It is intriguing that PS fibers with various grooved textures were obtained. The grooved fibers obtained from the solution of 10%, 15%, 20%, 25%, and 30% (*w*/*v*) concentrations had average diameters of 864, 1,704, 2,001, 2,040, and 2,570 nm, respectively (Figure 2A,B,C,D,E). The number of grooves declined from 5 to 7 at concentrations of 10% and 15% (*w*/*v*), to 3 to 4 at 20% and 25% (*w*/*v*), ending at just 1 groove at 30% (*w*/*v*). The depths of grooves at the concentrations of 10% and 15% (*w*/*v*) were relatively deeper than those of grooved fibers obtained from other concentrations. The average width between adjacent grooves of PS nanofibers obtained from 10% (*w*/*v*) was about 273 nm. Interestingly, these fibers are porous in the interior (Figure 3C,D and Figure 4). A plausible mechanism for this structure should be vapor-induced phase separation (VIPS), which is attributed to the mutual diffusion and penetration of THF, DMF, and water vapors [12]. In this case, water precipitates the polymer out of the solution to generate the solid matrix, whereas the solvent-rich phase evolves into porous regions.

**Figure 5:**
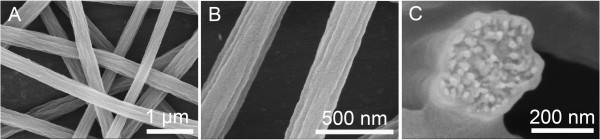
**SEM pictures of grooved nanofibers electrospun from 10% PS solution. (A, B)** Grooved nanofibers and **(C)** cross section. THF/DMF ratio 1:1 *v*/*v*, RH 40%, collecting distance 15 cm, feeding rate 1.5 ml/h, and applied voltage 12 kV.

In order to control the secondary structure as well as the diameter of grooved nanofibers, we also investigated other process parameters using 10% (*w*/*v*) PS solution (THF/DMF ratio, 1:1 *v*/*v*). Overall, applied voltage, collecting distance, and feeding rate had little effect on the secondary morphology and fiber diameter, but relative humidity exerted great influence on diameter of grooved PS nanofibers. Figure 5 shows the beaded free PS nanofibers obtained under a relative humidity of 40%. Inspiringly, the average diameter was only 326 ± 50 nm, and there were six to eight grooves well distributed along the axis of nanofibers. To the best of our knowledge, the average diameter of electrospun PS fibers was usually more than 1 μm, so these were the finest grooved nanofibers reported until now. The sharp decreased diameter of grooved nanofibers may be due to the lower relative humidity [22]. In this case, a relatively smaller amount of water diffused into the solution jet causes a delayed solidification, then leaving enough time for the jet to elongate due to Coulomb forces and whipping instability during traveling to the collector. Hence, grooved PS nanofibers with finer diameter are expected.

### Exploration of the formation mechanism of grooved texture

**Figure 6:**
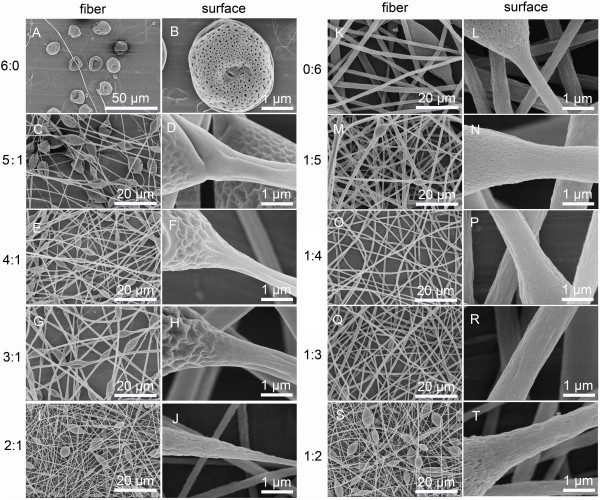
**SEM pictures of nanofibers and their surfaces fabricated by electrospinning 10% (*w*/*v*) PS solutions with various THF/DMF ratios. (A**, **B)** 6:0, **(C**, **D)** 5:1, **(E**, **F)** 4:1, **(G**, **H)** 3:1, **(I**, **J)** 2:1, **(K**, **L)** 0:6, **(M**, **N)** 1:5, **(O**, **P)** 1:4, **(Q**, **R)** 1:3, and **(S**, **T)** 1:2, *v*/*v*. RH 60%, collecting distance 15 cm, feeding rate 1.5 ml/h, and applied voltage 12 kV.

**Figure 7:**
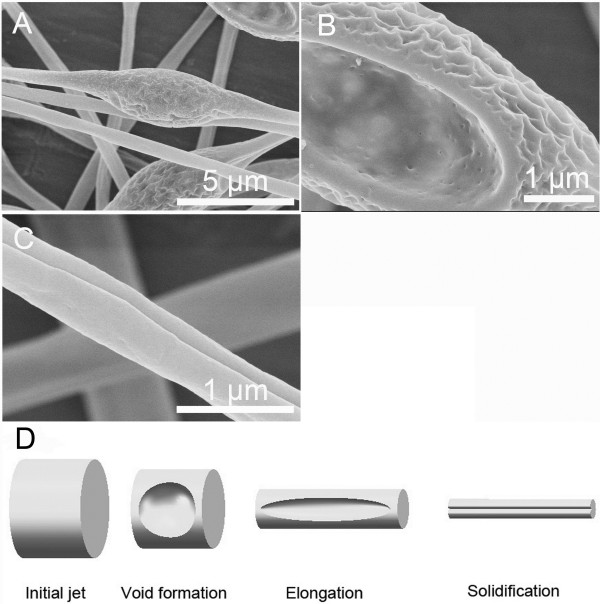
**Electrospun fibers and formation mechanism. (A, B, C)** Representative images of fibers electrospun from 10% (*w*/*v*) PS solution (THF/DMF ratio 4:1 *v*/*v*). RH 60%, collecting distance 15 cm, feeding rate 1.5 ml/h, and applied voltage 12 kV. **(D)** Formation mechanism of single grooved texture.

Figure 6 shows the morphology of nanofibers electrospun from 10% (*w*/*v*) PS solutions with various THF/DMF ratios. Bowl-like beads were obtained using pure THF as solvent. The outer surface of the bowl was porous, which is similar to nanofibers electrospun from 20% (*w*/*v*) PS/THF solution. Beaded fibers were formed when THF/DMF ratio was no less than 2:1 (*v*/*v*), it should be pointed out that nearly every bead had an elongated large void on the surface when THF/DMF ratio was higher than 2:1 (*v*/*v*), and most nanofibers between beads were single grooved (Figure 6C,D,E,F,G,H and Figure 7A,B,C). For the large void on the bead surface, the rapid evaporation of volatile THF (vapor pressure, 19.07 kPa) and subsequent transformation of the THF-rich region into voids could be the main reason. For single grooved texture, it should be attributed to the sufficient elongation of large voids when the jet was still incompletely solidified due to the residual solvent of less volatile DMF (vapor pressure, 0.36 kPa). When THF/DMF ratio was less than 1:2 (*v*/*v*), beaded nanofibers with a rough surface were produced, while the quantity of beads was less than that of nanofibers from larger THF/DMF ratios. As discussed before, when THF/DMF ratio was 1:1 (*v*/*v*), bead-free grooved nanofibers were obtained from 10% (*w*/*v*) PS solutions (Figures 2A and 5).

**Figure 8:**
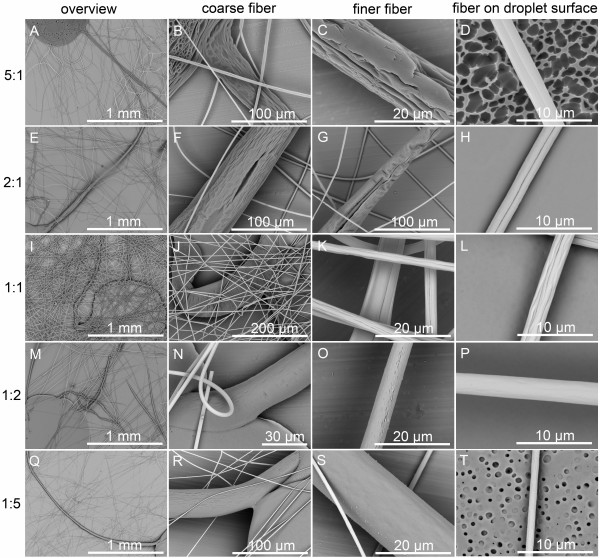
**SEM pictures of fibers and their surfaces from 20% (*w*/*v*) PS solutions with various THF/DMF ratios. (A**, **B**, **C**, **D)** 5:1, **(E**, **F**, **G**, **H)** 2:1, **(I**, **J**, **K**, **L)** 1:1, **(M**, **N**, **O**, **P)** 1:2, and **(Q**, **R**, **S**, **T)** 1:5, *v*/*v*. The solutions were electrospun under the lowest applied voltage. RH 60%, collecting distance 15 cm, feeding rate 1.5 ml/h, and applied voltage 5 kV.

Inspired by the cues from the electrospinning of 10% (*w*/*v*) PS solutions, 20% (*w*/*v*) PS solutions with various THF/DMF ratios were electrospun under the lowest applied voltage (5 kV). Therefore, fibers with insufficient elongation were expected to be obtained. It should be mentioned here that the process was unstable because the applied voltage was not high enough, so a glass rod had to be used to clean the tip of the needle and keep the setup working continuously. Interestingly, small droplets connected to coarse fibers can be produced from some of the solutions (THF/DMF ratios, 5:1, 2:1, 1:1, 1:2, 1:5 *v*/*v*), demonstrating the formation mechanism of grooved texture. The typical morphologies of the droplets and fibers are illustrated in Figure 8 and summarized in Table 1. When THF/DMF ratio was 5:1, numerous irregularly shaped pores in diameter of approximately 2 μm were found on the droplet surface, and the obtained fibers had a single grooved texture. In addition, there was a coarse fiber connected to the droplet, which has a diameter of 50 μm at the connection (exhibiting a grooved texture), while the diameter decreased to approximately 18 μm at the end of the coarse fiber. In this case, we can confirm that there were many large voids formed around the initial jet, so it is reasonable to assume that the formation of grooved texture should be attributed to elongation of large voids during electrospinning. Similarly, when THF/DMF ratio was 2:1, the coarse fiber with a diameter of 70 μm had a grooved texture, and the diameter decreased to approximately 20 μm at the end of the fiber. Even though no voids existed on the droplet surface, elongated voids (groove) presented on the surface of the coarse fiber, and all the resultant fibers were single grooved, which can also validate the aforementioned formation mechanism.

**Table 1:** Summary of the typical morphologies of the droplets and fibers

**THF/DMF ratio**	**Droplet**	**Coarse fiber**	**Finer fiber**	**Fibers**
5:1	Porous	Grooved	Grooved	Single grooved
2:1	Smooth	Single grooved	Single grooved	Single grooved
1:1	Smooth	Wrinkled	Grooved	Grooved
1:2	Smooth	Smooth	Smooth	Smooth
1:5	Porous	Smooth	Smooth	Smooth

When THF/DMF ratio was 1:1, no voids were found on the droplet surface and the coarse fiber at the connection appeared as a wrinkled surface, which resulted in a grooved texture at the end of the coarse fiber. In this case, we should attribute the formation of grooved texture to the wrinkled surface formed on the initial jet. When THF/DMF ratio was 1:2, both droplets and fibers had a smooth surface. Further reducing the ratio to 1:5, fibers having a smooth surface were observed, even though the droplet showed a porous surface.

**Figure 9:**
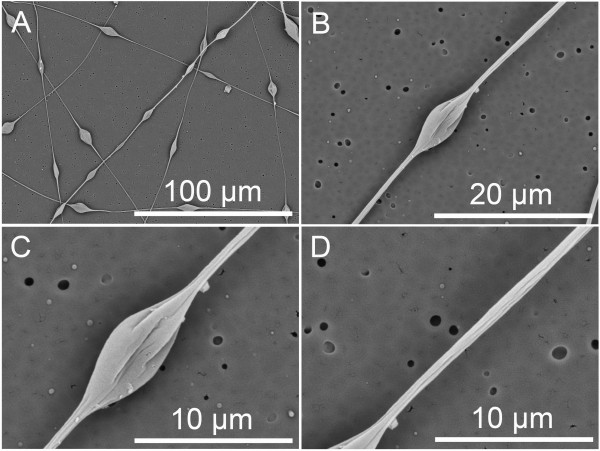
**SEM pictures of fibers and their surfaces from 10% (*w*/*v*) PS solutions (THF/DMF ratio 1:1** ***v*/*v*).** The solutions were electrospun under the lowest applied voltage. **(A)** Beaded nanofibers. **(B**, **C)** Bead. **(D)** Nanofiber. RH 60%, collecting distance 15 cm, feeding rate 1.5 ml/h, and applied voltage 5 kV.

To further investigate the formation mechanism of grooved texture, 10% (*w*/*v*) PS solutions (THF/DMF ratio, 1:1 *v*/*v*) were electrospun under the applied voltage of 5 kV. It is intriguing that both porous droplets and beaded fibers were produced. However, there were no voids but wrinkles on the surface of beads, while the nanofibers between beads also exhibited a grooved texture (Figure 9).

Based on the electrospinning results, we proposed that the formation mechanism of grooved texture should be attributed to two possible hypotheses. When THF/DMF ratio was higher than 2:1, as schematically illustrated in Figure 7D, the formation mechanism should be attributed to the formation of voids on the jet surface at the early stage of electrospinning and subsequent elongation and solidification of the voids into a line surface structure (mechanism I) [15]. This hypothesis can be supported by Figure 1C,D,E,F,G,H, Figure 6C,D,E,F,G,H, Figure 7A,B,C, and Figure 8A,B,C,D,E,F,G,H. Concerning fibers from 10% (*w*/*v*) PS solutions (THF/DMF ratios, 5:1, 4:1, 3:1 *v*/*v*), though there were wrinkles on the surface of void beads, the fibers between beads were single grooved, indicating that the formation of grooved texture should be attributed to voids but not wrinkles when THF/DMF ratio was higher than 2:1 (Figure 6C,D,E,F,G,H, Figure 7A,B,C). The main reason for the formation of voids on the initial jet surface should be ascribed to the higher volume ratio of THF and its higher volatility (vapor pressure, 19.07 kPa), which facilitates the rapid evaporation of THF and subsequent transformation of THF-rich region into voids. The less volume ratio of DMF and its lower volatility (vapor pressure, 0.36 kPa) should be another key factor to the formation of grooved texture [15]. During the formation of grooves, it is the residual DMF that kept the jet wet, which facilitates the void surface jet to be stretched into a grooved texture.

When THF/DMF ratio was 1:1, the formation mechanism should be ascribed to the formation of wrinkled surface on the jet surface at the early stage of electrospinning and subsequent elongation into a line surface structure (mechanism II). This hypothesis can be supported by Figure 8I,J,K,L and Figure 9A,B,C,D. In this case, THF and DMF can cooperate well with each other, the rapid evaporation of THF leads to the formation of semi-solidified shell on the initial jet surface, then the wrinkled surface was formed due to buckling of a cylindrical polymer shell under compressive radial stresses, arising from removal of the solvent from the core of the jet [21], while the residual DMF kept the jet wet, which facilitates the wrinkled surface jets to be stretched into a grooved texture.

To find more evidences of the formation mechanism of grooved texture, we also observed the interior structure of PS fibers with different surface morphologies (summarized in Table 2). Figure 3 shows the interior structure of PS fibers from 20% (*w*/*v*) with various THF/DMF ratios (4:1, 1:1, 0:6, *v*/*v*). When THF/DMF ratio was 4:1, the obtained fibers exhibited a heart-shaped cross section and solid interior structure, indicating that the formation of single grooved texture should be ascribed to mechanism I. When THF/DMF ratio was 1:1, the obtained fibers have a sawtooth cross section and porous interior structure; the corresponding fibers have a grooved surface. When THF/DMF ratio was 0:6, the obtained fibers have a circular cross section and porous interior structure, and no wrinkles or grooves can be found on the corresponding fibers surface even though the interior structure was porous, suggesting the indispensible role THF plays during the formation of grooved texture.

**Table 2:** Interior structure of PS fibers with different surface morphologies

**Concentration (%)**	**THF/DMF ratio**	**Interior structure**	**Cross section**	**Morphology**
20	4:1	Solid	Heart-like	Single grooved
20	1:1	Porous	Sawtooth	Grooved
20	0:6	Porous	Circular	Smooth
10	1:1	Porous	Sawtooth	Grooved
30	1:1	Porous	Heart-like	Single grooved

Figure 4 shows the interior structure of PS fibers from various solution concentrations with THF/DMF ratio 1:1 *v*/*v*. When the concentration was equal or less than 25% (*w*/*v*), the interior structures were similar to those at 20% (*w*/*v*). However, when the concentration was 30% (*w*/*v*), the obtained fibers have a heart-shaped cross section and porous interior structure. All fibers from PS solution (THF/DMF ratio, 1:1 *v*/*v*) exhibited a thin shell, which can provide sufficient support to hypotheses of formation mechanism II (Figure 3C,D, Figure 4, and Figure 5C).

## Conclusion

We have demonstrated a convenient and reliable method to fabricate grooved PS nanofibers. The average diameter of the grooved nanofibers was as small as 326 ± 50 nm, and we believe they are so far the finest nanofibers with a grooved texture. By systematical investigation of process parameters, we pointed out that solvent system, solution concentration, and relative humidity were the three key factors to the formation of grooved texture. When THF/DMF ratio was higher than 2:1, the formation mechanism should be attributed to the formation of voids on the jet surface at the early stage of electrospinning and subsequent elongation and solidification of the voids into a line surface structure. When THF/DMF ratio was 1:1, the formation mechanism should be ascribed to the formation of wrinkled surface on the jet surface at the early stage of electrospinning and subsequent elongation into a grooved texture.

## Abbreviations

CAB: cellulose acetate butyrate; DMF: *N*,*N*-dimethylformamide; FE-SEM: field emission scanning electron microscopy; PS: polystyrene; RH: relative humidity; THF: tetrahydrofuran; *v*/*v*: volume/volume; *w*/*v*: weight (g)/volume (ml).

## Competing interests

The authors declare that they have no competing interests.

## Authors’ contributions

WL designed and performed the experimental work and explained the obtained results and wrote the paper. CH and XJ helped in writing of the paper and participated in the experimental work. All authors read and approved the final manuscript.
